# Depression and peripheral artery disease: why we should care and what we can do

**DOI:** 10.1186/s42155-018-0017-1

**Published:** 2018-09-18

**Authors:** Joel L. Ramirez, S. Marlene Grenon

**Affiliations:** 1 Department of Surgery, Division of Vascular and Endovascular Surgery, University of California, San Francisco, CA USA; 2 Vascular Surgery Section, Veterans Affairs Medical Center, Mail Code 112G, 4150 Clement St, San Francisco, CA 94121 USA

To the Editor

Peripheral artery disease (PAD) is a global problem that has been estimated to affect more than 200 million people worldwide with increasing disease incidence (Fowkes et al. 2013). Patients with PAD experience a dramatically high risk of adverse cardiovascular events and mortality, as well as impaired quality of life due to claudication symptoms, limb loss, and physical disability. Although several traditional risk factors for PAD have been identified, a more comprehensive understanding of the factors that may influence the development and progression of PAD is essential in order to adequately care for this high-risk patient population.

Recent evidence suggests that depression may represent an under-recognized risk factor for PAD. Although the relationship between depression and other atherosclerotic diseases such as coronary artery disease (CAD) is better understood and supported by robust evidence (Elderon and Whooley 2013), our understanding of how depression may affect PAD remains more limited. However, previous evidence linking depression with CAD suggests several plausible mechanistic pathways through which depression may be associated with PAD. These mechanisms include dysregulation of the hypothalamic-pituitary-adrenal axis, autonomic system, immune system, endothelial function, and coagulation cascade, as well as an increased prevalence of several behavioral risk factors associated with atherogenesis, including tobacco use, physical inactivity, and medical non-adherence (Ramirez et al. 2018).

Recent work focused on patients with PAD has begun to highlight the impact that depression may have on this patient population (Ramirez et al. 2018). Patients in the community who develop PAD have been reported to have a higher prevalence of depressive symptoms, and diagnoses of depression have been associated with incident PAD (Wattanakit et al. 2005). McDermott et al. reported in a cross-sectional analysis of The Walking and Leg Circulation Study the presence of depression or depressive symptoms in 19.6% of patients with PAD, but only in 13.2% of patients without PAD (*p* = 0.003) (McDermott et al. 2016). Among patients with pre-existing PAD, depression has been associated with impaired physical function (Smolderen et al. 2008; McDermott et al. 2003; Ruo et al. 2007), increased mortality (McDermott et al. 2016), worse revascularization patency and recurrence of symptoms after peripheral revascularization (Cherr et al. 2007), increased risk of major amputation (Arya et al. 2018), and increased progression of PAD (Cherr et al. 2008). However, patients with pre-existing PAD are also more likely to develop depressive symptoms (McDermott et al. 2016), suggesting that the relationship between PAD and depression may be bi-directional.

Although the directionality of this relationship remains unclear, the evidence linking these two diseases is undeniable. As endovascular and vascular specialists, we carry the responsibility of treating and preventing PAD. As such, recognizing depression as an important risk factor for poor outcomes in patients with PAD is essential to providing the highest quality care. Although there are currently no guidelines available that address the screening, diagnosis, and management of depression among patients with PAD, guidelines do exist for patients with CAD (Lichtman et al. 2008) and may be generalizable to those with PAD. These guidelines include screening for depression using the two or nine item Patient Health Questionnaire (PHQ), and referring patients to a mental health specialist when appropriate. However, no studies to date have examined the effect of treating depression on the progression of PAD or outcomes among patients with PAD.

Further research is warranted to more comprehensively understand the relationship between depression and PAD. This includes an understanding of depression’s physiologic implications on peripheral atherogenesis, vascular injury, and recovery after surgical or endovascular interventions. We strongly encourage investigators conducting research in patients with PAD to collect information on mental health comorbidities and treatment. We recommend collecting data related to mental health diagnoses and/or eliciting a brief psychiatric history, documenting psychiatric medications, and screening for depression using validated questionnaires, such as the PHQ-9. Doing so would not only provide valuable evidence to further characterize the relationship between depression and PAD, but also has the potential to aid in the development of management guidelines to improve the outcomes of patients with comorbid depression and PAD. As the global population ages and the incidence of PAD increases, properly recognizing and treating all of the risk factors associated with PAD will become essential to reducing the burden of this disease.
